# Hsa_circ_0007312 Promotes Third-Generation Epidermal Growth Factor Receptor-Tyrosine Kinase Inhibitor Resistance through Pyroptosis and Apoptosis via the MiR-764/MAPK1 Axis in Lung Adenocarcinoma Cells

**DOI:** 10.7150/jca.72066

**Published:** 2022-06-27

**Authors:** Chenyue Dai, Zeming Ma, Jiahui Si, Guo An, Wenlong Zhang, Shaolei Li, Yuanyuan Ma

**Affiliations:** 1 Department of Thoracic Surgery II, Key Laboratory of Carcinogenesis and Translational Research (Ministry of Education), Peking University Cancer Hospital and Institute, Beijing, China; 2 Department of Anesthesiology, Key Laboratory of Carcinogenesis and Translational Research (Ministry of Education), Peking University Cancer Hospital and Institute, Beijing, China; 3 Department of Laboratory Animals, Key Laboratory of Carcinogenesis and Translational Research (Ministry of Education), Peking University Cancer Hospital and Institute, Beijing, China

**Keywords:** lung adenocarcinoma, epidermal growth factor receptor-tyrosine kinase inhibitor resistance, circular RNA, pyroptosis

## Abstract

**Purposes:** Osimertinib is a third-generation epidermal growth factor receptor-tyrosine kinase inhibitor (EGFR-TKI) used for patients with gefitinib (first-generation EGFR-TKI) resistance, but osimertinib resistance inevitably occurs. Therefore, it is necessary to explore the mechanisms of osimertinib resistance.

**Materials and Methods:** We performed quantitative real-time polymerase chain reaction to detect hsa_circ_0007312 (circ7312), miR-764, and MAPK1 expressions in tissues and cells. Western blotting was used to detect protein levels in cells. Cell Counting Kit-8, apoptotic, and Transwell assays were used to explore biological functions. Luciferase assays were used to identify the interactions between circ7312 and miR-764, MAPK1 and miR-764. A xenograft experiment was performed to clarify the role of circ7312 *in vivo*. Public datasets were used to identify the relation between circ7312 expression and the cell half maximal inhibitory concentration value of osimertinib in 41 lung adenocarcinoma cell lines. The Student t-test, Kaplan-Meier analysis, and Pearson correlation analysis were used in data analysis.

**Results:** We found that circ7312 knockdown increased miR-764 expression and decreased MAPK1 expression, and circ7312 regulated MAPK1 by sponging miR-764. In addition, high circ7312 expression has significant positive correlation with osimertinib IC50 values, circ7312 knockdown decreased the cell half maximal inhibitory concentration value of osimertinib and increased pyroptosis and apoptosis by sponging the miR-764/MAPK1 axis. We also found that circ7312 and MAPK1 were highly expressed in tumor tissues and related to poor prognosis. Xenograft experiments revealed that circ7312 knockdown decreased osimertinib resistance *in vivo*.

**Conclusion:** We demonstrated that the inhibition of circ7312 decreased osimertinib resistance by promoting pyroptosis and apoptosis via the miR-764/MAPK1 axis, providing a novel target for osimertinib resistance therapy.

## Introduction

Lung cancer is the leading cause of cancer-related mortality worldwide in both sexes, with lung adenocarcinoma (LUAD) being the most prevalent histological subtype 1, 2. Third-generation epidermal growth factor receptor-tyrosine kinase inhibitors (EGFR-TKIs), such as osimertinib, have been recommended as the standard therapy for LUAD patients with acquired first-generation EGFR-TKI resistance; although osimertinib improved disease-free survival (DFS) and overall survival (OS), acquired resistance to osimertinib developed 3, 4. The mechanisms of acquired resistance to EGFR-TKIs mainly include the modification of target genes, activation of bypass signaling pathways, and phenotypic transformation 5, 6. Recently, the effect of programmed cell death (PCD) including apoptosis, pyroptosis, and autophagy on drug resistance has received more attention 7-9. Thus, we further explored the underlying role of PCD in mechanisms of third-generation EGFR-TKI resistance.

Emerging studies have clarified that pyroptosis and apoptosis are involved in drug resistance, either alone or in combination. Zhang et al. reported that paclitaxel and cisplatin activated the caspase3/Gasdermin E (GSDME) pathway to induce pyroptosis in A549 cells, and Xu et al. indicated that downregulation of miR-155-5p can enhance apoptosis and pyroptosis to improve the anti-tumor effect of cetuximab^10^, ^11^. Pyroptosis can be activated by the classic caspase-1-dependent pathway and non-classic caspase-4/5/11-dependent pathway, then activated by caspase-cleaved Gasdermin D (GSDMD) to trigger pyroptosis^12^. Caspase-3 was found to cleave GSDME to induce pyroptosis^13^. However, the role of pyroptosis in osimertinib resistance is unclear, so we hypothesized that pyroptosis would be involved in osimertinib resistance and performed a series of functional assays to clarify this mechanism.

Circular RNAs (circRNAs) have been identified that can serve as oncogenes or tumor suppressor genes to regulate the development of various cancers, and are involved in anti-cancer drug resistance through different molecular mechanisms, including microRNA (miRNA) sponging, protein interaction, and transcriptional or post-transcriptional regulation 14, 15. Moreover, the circRNA-miRNA-messenger RNA regulatory network was found to play a vital role in chemotherapy resistance and targeted therapy resistance 16. For instance, circ-CPA4 was identified to promote chemoresistance by acting as a let-7 miRNA sponge 17. In addition, hsa_circ_0004015 was found to enhance EGFR-TKI resistance through the miR-1183/PDPK1 signaling pathway 18.

In our previous study, we found that circ7312 and MAPK1 were highly expressed in EGFR-TKI-resistant PC9 cells but were lowly expressed in the EGFR-TKI-sensitive PC9/ER cells. We also found that the circ7312/miR-764/MAKP1 axis is involved in the development of EGFR-TKI resistance 19. Therefore, in this study, we aimed to determine the underlying mechanism of this axis in pyroptosis and apoptosis that is involved in EGFR-TKI resistance.

## Materials and Methods

### Ethics statements

This study was approved by the Ethics Committee of Peking University Cancer Hospital (Beijing, China) and conducted in accordance with the Declaration of Helsinki. Written informed consent was obtained from all the participants prior to the study.

### Patients and tissues

The inclusion criteria for patients enrolled into our study included: (1) pathologically diagnosed with LUAD; (2) performed R0 resection; (3) no diagnosis of any other malignant diseases; (4) complete basic information, including age, gender, histology, TNM stage, and follow-up data. The exclusion criteria included one or more of the following: (1) R1 or R2 resection; (2) a history of other malignant diseases within 5 years. According to the inclusion and exclusion criteria, 89 paired tumor tissues and adjacent normal tissues were collected from patients with lung adenocarcinoma who underwent surgery in the Department of Thoracic Surgery Ⅱ of Peking University Cancer Hospital (Beijing, China) from December 2011 to December 2012, and all tissues were immediately stored in liquid nitrogen.

### Cell lines and cell culture

The EGFR-TKI-sensitive lung adenocarcinoma cell line PC9 and EGFR-TKI-resistant lung adenocarcinoma cell line PC9/ER were maintained in our laboratory. PC9/ER cells were generated from parental PC9 cells following exposure to gefitinib as we performed previously 20. PC9 cells and PC9/ER cells were cultured in an RPMI-1640 medium (Gibco BRL, Gaithersburg, MD, USA) supplemented with 10% fetal bovine serum (Gibco BRL) and 1% penicillin-streptomycin (Invitrogen, Waltham, MA, USA). All cells were incubated in a humidified atmosphere at 37°C with 5% carbon dioxide (CO_2_).

### Reagents and cell transfection

Small interfering RNA targeting circ-0007312 (si-circ7312), small interfering RNA negative control (si-control), mimic targeting miR-764 (mimic-mi764), mimic negative control (mimic-control), small interfering RNA targeting MAPK1 (si-MAPK1), and small interfering RNA negative control (si-control) were purchased from RiboBio (Guangzhou, China). Cells were seeded into a 6-well plate, and cell transfection was performed using Lipofectamine 3000 (Invitrogen) according to the manufacturer's instructions.

### Total RNA extraction and quantitative real-time polymerase chain reaction

Total RNA was extracted from tissues and cells using the TRIzol reagent (Invitrogen) according to the manufacturer's protocol. The RNA concentration was measured using Nanodrop (Thermo Fisher Scientific, Waltham, MA, USA). Complementary DNA (cDNA) synthesis was performed using the Easy Script First-Strand cDNA Synthesis Super Mix (Transgen Biotech, Beijing, China) according to the manufacturer's instructions. Reverse transcription of microRNA was performed using the TaqMan MicroRNA Reverse Transcription Kit (Applied Biosystems, Foster City, CA, USA). The relative expressions of circ7312, miR-764, and MAPK1 were detected by the 7500 Fast Real-Time PCR System (Thermo Fisher Scientific) using the Go Taq qPCR Master Mix (Promega Corporation, Madison, USA). GAPDH and U6 were used as internal controls to normalize the expression levels of circ7312, miR-764, and MAPK1. The 2^-ΔCt^ method was used in this process. The primers used in this study were as follows: hsa_circ_0007312, 5'-TAACGGTGACTAATGGTGTTAAAGG-3' (forward) and 5'-GATGCATTTACAAGCAAATCTGTGC-3'(reverse); hsa-miR-764, 5'-GCAGGTGCTCACTTGTCCTCCT-3' (forward) and 5'-GCGAGCACAGAATTAATACGAC-3' (reverse); and MAPK1, 5'-TGTTCCCAAATGCTGACT-3' (forward) and 5'-AACTTGAATGGTGCTTCG-3' (reverse).

### Western blotting

PC9 and PC9/ER cells were harvested and lysed using the RIPA lysis buffer (Solarbio, Beijing, China). Total proteins were quantified by the BCA assay. Equal amounts of protein were separated by 10% SDS-PAGE and transferred to polyvinylidene difluoride membranes. The membranes were blocked with 5% skim milk (Solarbio) for 1 hour at room temperature. The membranes were incubated with primary antibodies overnight at 4°C and then with secondary antibodies for 1 hour at room temperature. The membranes were visualized with chemiluminescence reagents (NCM Biotech, Suzhou, China) using the Amersham Imager 680 (GE Healthcare, Chicago, IL, USA). Primary antibodies including MAPK1, p-MAPK1, caspase-3, cleaved-caspase-3, and GAPDH were purchased from Cell Signaling Technology (Danvers, MA, USA). The DSDME antibody was purchased from Abcam (Cambridge, MA, USA). The GAPDH antibody served as the internal control.

### Cell Counting Kit-8 assay

Osimertinib (AZD9291) was purchased from Selleck Chemicals (Houston, TX, USA). PC9/ER cells (5×10^3^ cells/well) were seeded in 96-well plates for 24 hours, and osimertinib was added to the 96-well plates for 48 hours. Cell proliferation was monitored using the Cell Counting Kit-8 (CCK-8, Dojindo, Kumamoto, Japan), and the absorbance at 450 nm was measured using the Infinite 200 Pro (Tecan, Männedorf, Switzerland). The cell half maximal inhibitory concentration (IC50) was calculated using GraphPad software (San Diego, CA, USA).

### Transwell assay

The PC9/ER cells (1×10^5^ cells/well) were seeded in 24-well plates with a chamber; the upper chamber was filled with a serum-free medium, and the lower chamber was filled with the RPMI-1640 medium supplemented with 10% fetal bovine serum. For the migration assay, the upper chamber did not include Matrigel (BD Biosciences, Bedford MA, USA). For the invasion assay, the upper chamber included Matrigel. After 24 hours, the cells migrated or invaded through the upper chamber; 4% paraformaldehyde was used to fix the cells for 10 minutes, and 0.1% crystal violet was used to stain the cells for 5 minutes. An inverted microscope (Canon, Tokyo, Japan) was used to capture the images, and ImageJ software (National Institutes of Health, Bethesda, MD, USA) was used to calculate the number of cells.

### Dual-luciferase reporter assay

The circ7312 fragment containing the potential miR-764 binding sites and the fragment of MAPK1 containing the miR-764 binding sites were synthesized by Sangon Biotech (Beijing, China). The pmiR-RB-Report vector was purchased from RiboBio (Guangzhou, China). These two fragments were inserted into the pmiR-RB-Report vector. The firefly luciferase gene was used as the internal control gene, and the Renilla luciferase gene was used as the reporter gene. PC9/ER cells (1×10^5^ cells/well) were seeded in a 24-well plate and transferred with mimic-miR-764 and the pmiR-RB-Report vector for 48 hours. Luciferase activity was measured using the Dual-Luciferase Reporter Assay System (Promega, Madison, WI, USA).

### Apoptotic assay

The Annexin V FITC Apoptosis Detection Kit (Dojindo, Japan) was used to detect apoptotic cells according to the manufacturer's instructions. The treated cells were harvested and rinsed with phosphate-buffered saline (Gibco BRL). Propidium iodide staining and Annexin V-FITC staining were added to different groups of cell suspensions, and the cell suspension was incubated in the dark for 15 minutes at room temperature. The samples were examined using CytoFlex (Beckman Coulter, Brea, CA, USA). All data were analyzed using the FlowJo software (BD, Ashland, OR, USA).

### Xenograft experiment

Xenograft experiments were performed to identify the role of circ7312 *in vivo*. The animal experiments were approved by the Ethics Committee of the Animal Experiment of Peking University Cancer Hospital. In total, 2×10^6^ cells were subcutaneously injected into the backs of female BALB/C nude mice (Beijing HFK Bioscience, Beijing, China). The tumor volume was measured every 3 days and recorded. The volume was calculated as follows: volume=length×width^2^/2. When the tumor grew to approximately 100 mm^3^, the mice were randomly divided into two groups (n=5). The two groups were treated with osimertinib (0.2 mg per mouse) by oral gavage (5 days/week). One group was treated with si-circ7312 (5 nmol per mouse) via intratumoral injection every 4 days. The other group was treated with si-control and served as the control group. All reagents were synthesized by RiboBio. After 36 days, the BALB/C nude mice were sacrificed by CO^2^, and the tumors were resected and stored in liquid nitrogen.

### Statistical analysis

Osimertinib IC50 values data in cancer cell lines were downloaded from the Genomics of Drug Sensitivity in Cancer Project (https://www.cancerrxgene.org/) 21. Gene expression data in lung adenocarcinoma cell lines were downloaded from the Cancer Cell Line Encyclopedia (https://sites.broadinstitute.org/ccle/) 22. These two datasets were cleaned and matched according to cancer cell line names using R version 4.1.3 (https://www.r-project.org/). All experiments were performed in triplicate. The numerical numbers are presented as mean ± standard deviation. Differences between two groups were analyzed using the Student t-test. Survival analysis was performed using the Kaplan-Meier analysis and Pearson correlation analysis. Statistical significance was set at *P*<0.05.

## Results

### Circ7312 is highly expressed in lung adenocarcinoma tissues and related to a poor prognosis

**Figure 1:**
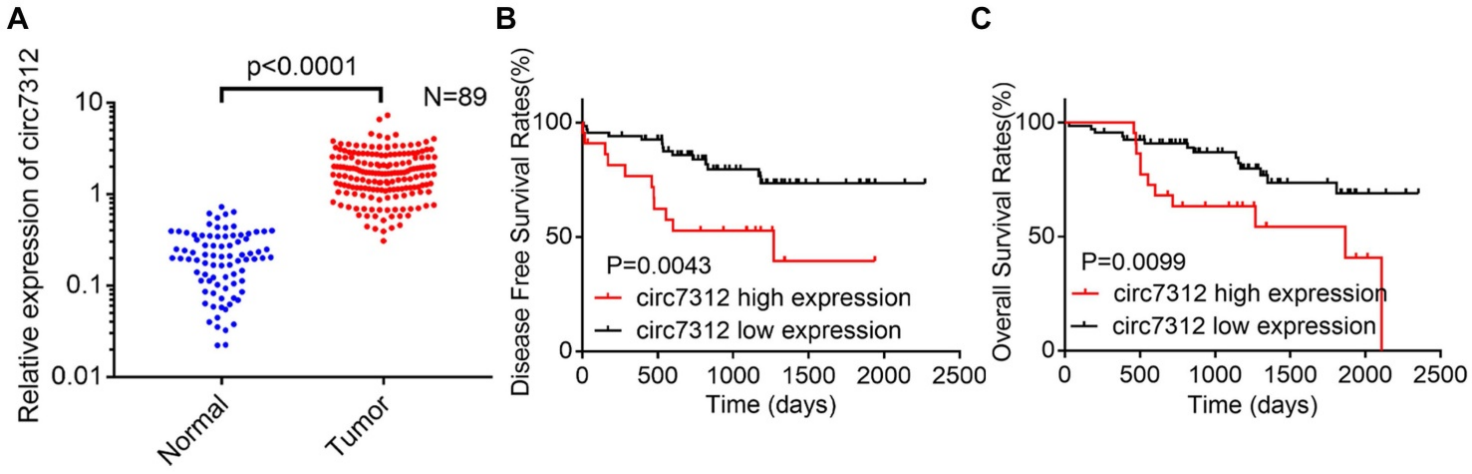
Circ7312 is highly expressed in lung adenocarcinoma (LUAD) tissues and related to poor prognosis. A. The relative expression of circ7312 is detected by qualitative real-time polymerase chain reaction in 89 LUAD tissues and paired normal tissues. B, C. Kaplan-Meier survival analysis shows the disease-free survival (B) and overall survival (C) between the high circ7312 expression and low circ7312 expression groups.

To clarify the role of circ7312 in LUAD, we detected the relative expression of circ7312 using qRT-PCR in 89 LUAD tissues and paired normal tissues. We found that circ7312 was upregulated in tumor tissues compared to that in normal tissues, as shown in Figure 1A. DFS and OS were significantly longer in LUAD patients with low circ7312 than in those with high circ7312 expression (Figure 1B, C). Statistical analysis indicated that the expression of circ7312 was associated with the TNM stage and 5-year survival (Table 1). These data indicate that high circ7312 expression is related to poor prognosis.

### High circ7312 expression has significant positive correlation with osimertinib IC50 values

To further investigate the role of circ7312 in osimertinib response, the relation between circ7312 expression level and osimertinib IC50 values from public datasets including the Genomics of Drug Sensitivity in Cancer Project and the Cancer Cell Line Encyclopedia were analyzed. Circ7312 expression level and IC50 value of osimertinib were identified in the 41 lung adenocarcinoma cell lines (Table S1). We found the expression level of circ7312 had significant positive correlation with osimertinib IC50 values (Figure S1), indicating that high circ7312 expression is related to osimertinib resistance.

### Osimertinib resistance is associated with pyroptosis and apoptosis

**Figure 2:**
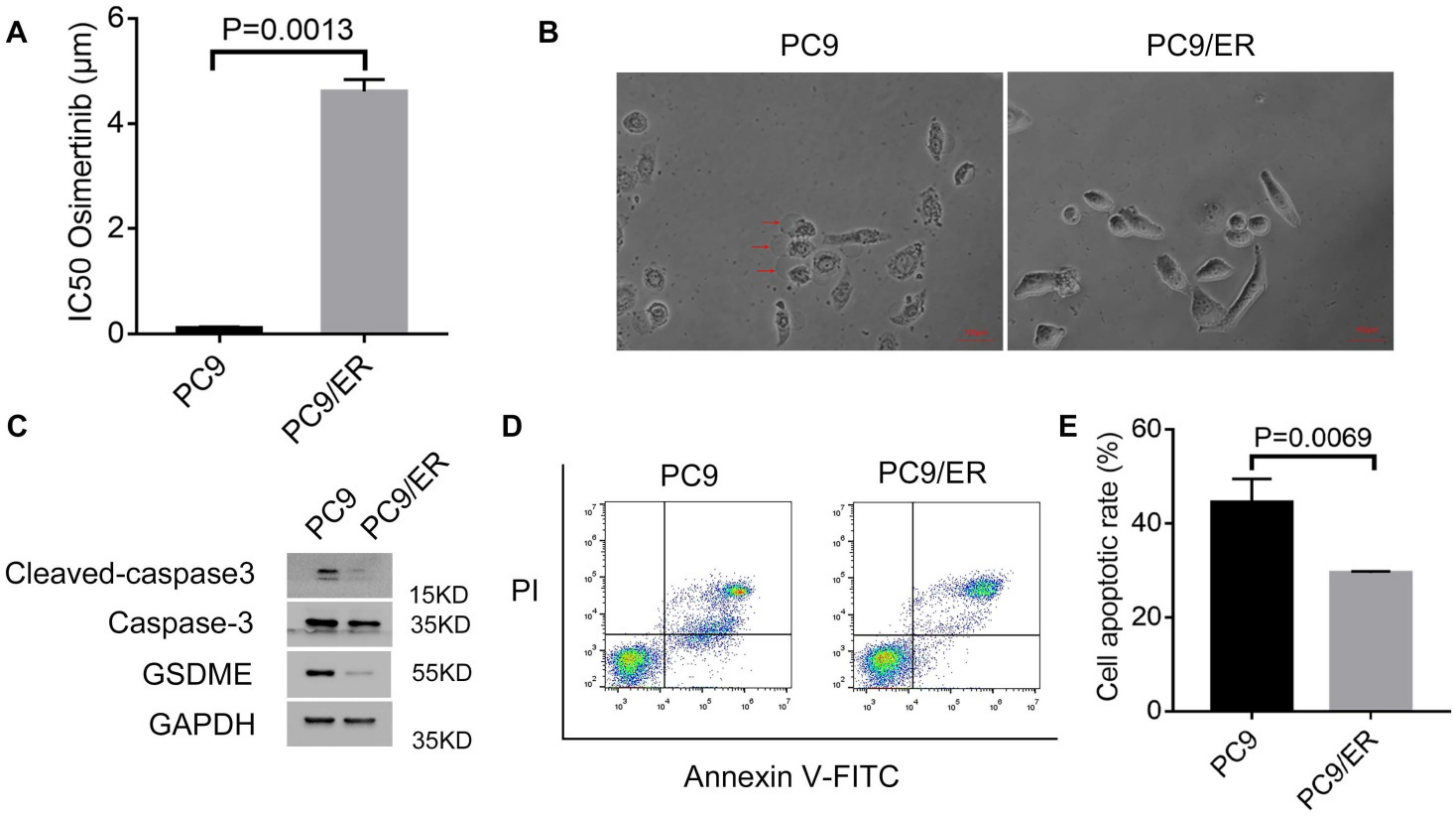
Osimertinib resistance is associated with pyroptosis and apoptosis. A. Half-maximal inhibitory concentration (IC50) values of osimertinib in PC9 and PC9/ER cells are measured by Cell Counting Kit-8 assays. B. Representative images of PC9 and PC9/ER cells with treatment of osimertinib (1 μmol) for 12 hours. The red arrows indicate the dying cells swelling with large bubbles. Scare bar, 100 μm. C. Western blotting analysis of GSDME, caspase-3, and cleaved caspase-3 protein expressions in PC9 and PC9/ER cells with treatment of osimertinib (1 μmol) for 48 hours. D, E. The apoptotic rate is measured using an Annexin V FITC/propidium iodide double staining in PC9 and PC9/ER cells treated with osimertinib (1 μmol) for 48 hours (D). The histogram shows the different apoptotic rates (E).

In our previous study, we found that circ7312 was upregulated in PC9/ER cells compared to PC9 cells. We examined IC50 in PC9 and PC9/ER cells and found that the IC50 values of osimertinib in PC9/ER cells significantly increased (Figure 2A). We exposed PC9 cells and PC9/ER cells with osimertinib and observed large bubbles blowing from the membrane in PC9 cells, which is a typical morphology of pyroptosis (Figure 2B). Subsequently, we exposed PC9 and PC9/ER cells with osimertinib and found that the expression of pyroptosis-related proteins including GSDME, caspase-3, and cleaved caspase-3 were highly expressed in PC9 cells (Figure 2C). We also performed apoptotic assays to test the apoptotic rate in PC9 and PC9/ER cells treated with osimertinib, and the results showed a higher apoptotic rate in PC9 cells than in PC9/ER cells (Figure 2D, E). These results demonstrate that the reduction of pyroptosis and apoptosis contributed to osimertinib resistance in PC9/ER cells.

### Circ7312 knockdown decreases osimertinib resistance and increases pyroptosis and apoptosis

**Figure 3:**
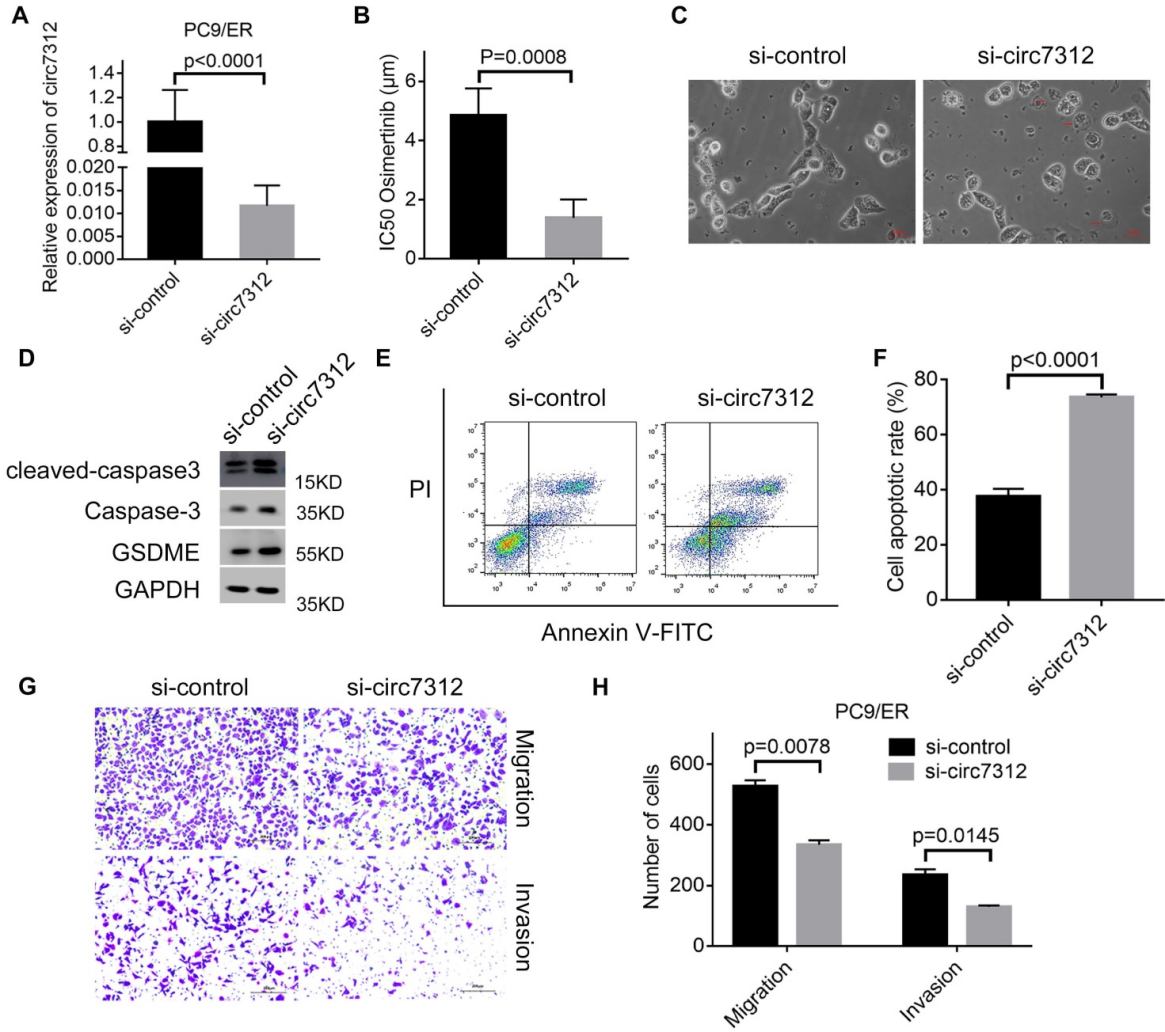
Circ7312 knockdown decreases osimertinib resistance and increases pyroptosis and apoptosis. A. The transfection efficiency of small interfering RNA targeting circ-0007312 (si-circ7312) is examined after 48 hours by qualitative real-time polymerase chain reaction. B. Cell Counting Kit-8 assays are performed to measure the half-maximal inhibitory concentration (IC50) of osimertinib in circ7312 knockdown PC9/ER cells treated with osimertinib. C. Representative images of dying circ7312 knockdown PC9/ER cells treated with osimertinib (1 μmol) for 12 hours. The red arrows indicate the cells swelling with large bubbles. Scare bar, 100 μm. D. Western blotting analysis of GSDME, caspase-3, and cleaved caspase-3 expressions in circ7312 knockdown PC9/ER cells treated with osimertinib (1 μmol) for 48 hours. GAPDH is used as an internal control. E, F. Apoptotic assays are performed to identify the apoptotic rate in circ7312 knockdown PC9/ER cells treated with osimertinib (1 μmol) for 48 hours (E). The histogram shows the different apoptotic rates (F). G, H. Transwell assays are performed to examine the migration and invasion capacity in circ7312 knockdown PC9/ER cells (G) Scare bar, 200 μm. The histogram shows the numbers of migration and invasion cells (H).

To clarify the potential role of circ7312 in osimertinib resistance, we transfected PC9/ER cells with si-circ7312, and the transfection efficiency was determined by qRT-PCR (Figure 3A). Subsequently, the transfected PC9/ER cells were exposed to osimertinib, and CCK-8 assays showed that circ7312 knockdown significantly decreased the IC50 values of osimertinib in PC9/ER cells (Figure 3B). We observed the formation of bubbles in circ7312 knockdown PC9/ER cells treated with osimertinib (Figure 3C). The expression of pyroptosis-relevant proteins including GSDME, caspase-3, and cleaved caspase-3 was increased in circ7312 knockdown PC9/ER cells treated with osimertinib (Figure 3D). We found a higher apoptotic rate in circ7312 knockdown PC9/ER cells treated with osimertinib than in those transfected with si-control (Figure 3E, F). In addition, we demonstrated that circ7312 knockdown decreased the migration and invasion abilities of PC9/ER cells (Figure 3G, H). These results all implied that circ7312-promoted osimertinib resistance may be regulated by pyroptosis and apoptosis processes.

### MiR-764 is sponged by circ7312 in osimertinib-resistant cells

**Figure 4:**
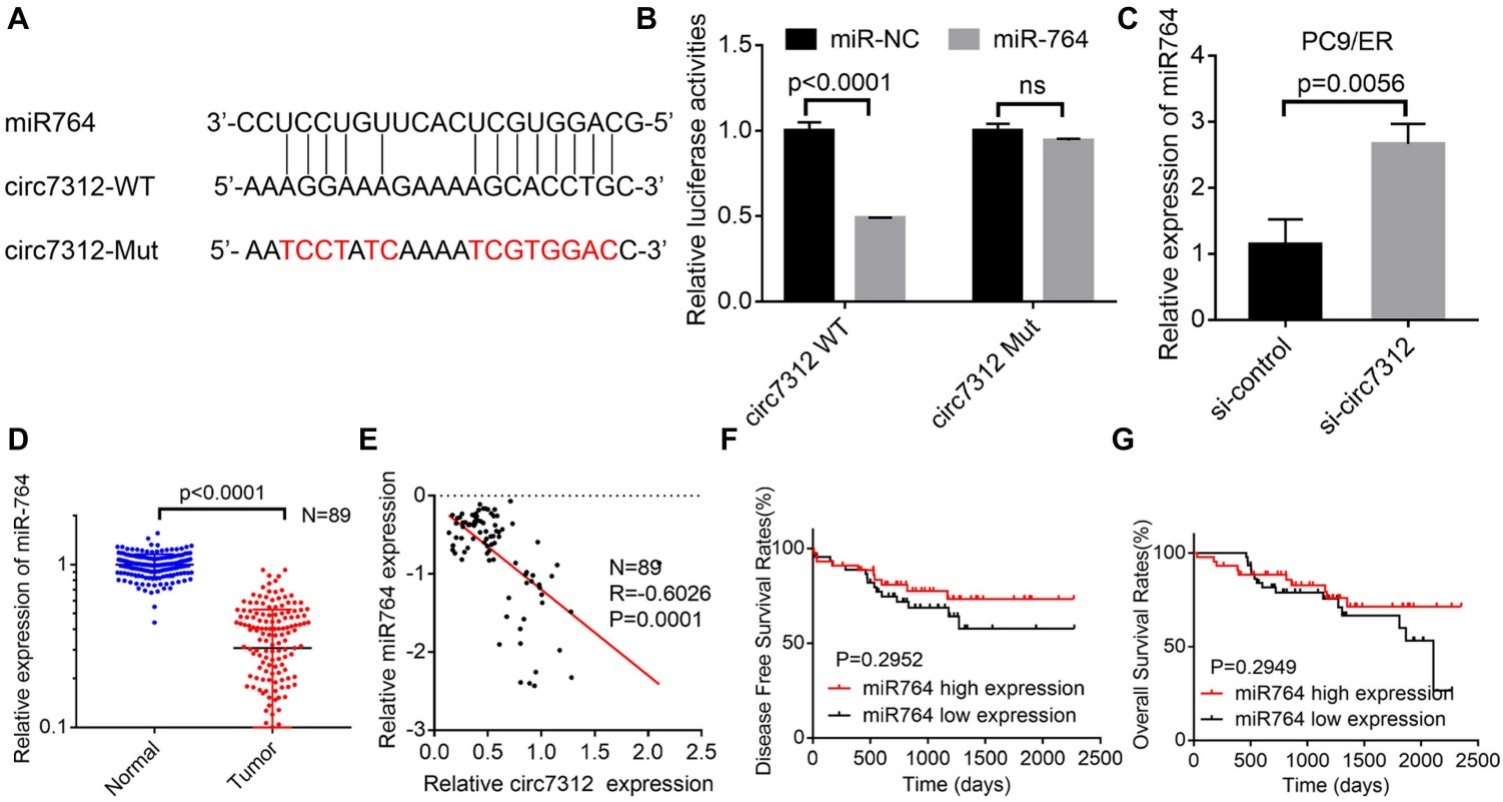
MiR-764 is sponged by circ7312 in osimertinib-resistant cells. A. The sequence of the miR-764 and circ7312 fragment including potential binding sites with miR-764 is predicted and synthesized. B. Luciferase assays are performed to identify the interrelationship between circ7312 and miR-764. C. The relative expression of miR-764 is examined by qualitative real-time polymerase chain reaction (qRT-PCR) in circ7312 knockdown PC9/ER cells. D. The relative expression of miR-764 is detected by qRT-PCR in 89 lung adenocarcinoma tissues and paired normal tissues. E. Spearman correlation analysis shows the negative correlation between circ7312 and miR-764. F, G. Kaplan-Maier survival analysis clarifies the disease-free survival (F) and overall survival (G) between the high miR-764 expression and low miR-764 expression groups.

We demonstrated that circ7312 can act as an miR-764 sponge based on bioinformatics analysis. To further verify whether circ7312 indeed targeted miR-764, we predicted the binding site between circ7312 and miR-764 (Figure 4A). Co-transfection of circ7312-WT and mimic-miR-764 increased luciferase activity compared to circ7312-Mut and mimic-miR-764 (Figure 4B). Knockdown of circ7312 upregulated the expression of miR-764 (Figure 4C). In addition, we detected the expression of miR-764 in 89 LUAD tissues and paired normal tissues and found that miR-764 was lowly expressed in tumor tissues (Figure 4D). Spearman correlation analysis confirmed a negative correlation between circ7312 and miR-764 (Figure 4E). Furthermore, we observed that there was no significant difference in DFS and OS between the high miR-764 expression and low miR-764 expression groups (Figure 4F, G). These results indicated a negative correlation between circ7312 and miR-764, indicating the role of circ7312 in sponging miR-764.

### MiR-764 suppresses osimertinib resistance and promotes pyroptosis and apoptosis

**Figure 5:**
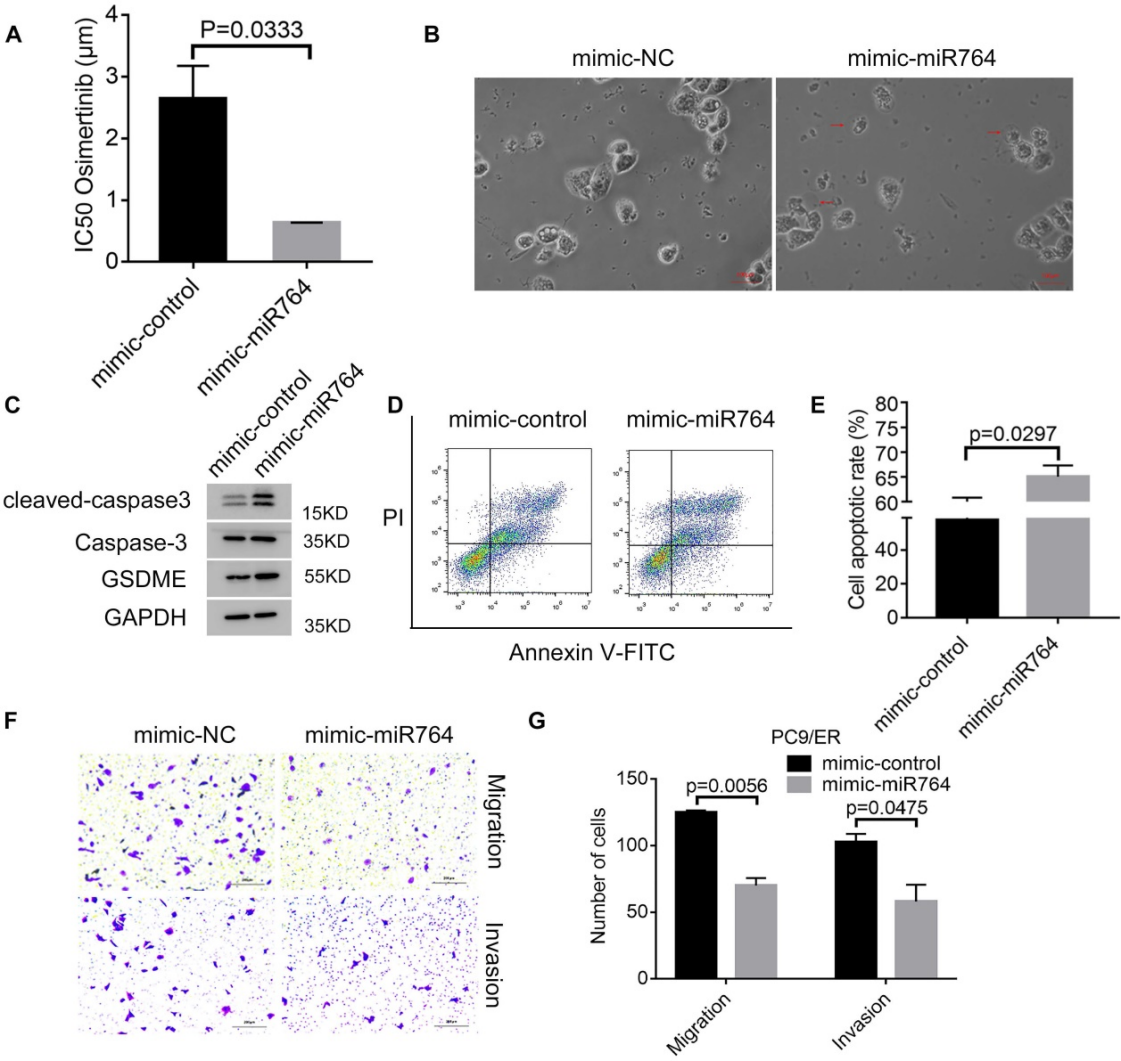
MiR-764 suppresses osimertinib resistance and promotes pyroptosis and apoptosis. A. Cell Counting Kit-8 assays are performed to identify the half-maximal inhibitory concentration (IC50) values of osimertinib in miR-764 overexpression PC9/ER cells. B. The representative images of dying miR-764 overexpression PC9/ER cells treated with osimertinib (1 μmol) for 12 hours. The red arrows indicate the cell swelling with large bubbles. Scare bar, 100 μm. C. Western blotting analysis shows the protein expression of GSDME, caspase-3, and cleaved caspase-3 in miR-764 overexpression PC9/ER cells treated with osimertinib (1 μmol) for 48 hours. D, E. The apoptotic rate is measured using Annexin V-FITC/PI double staining in miR-764 overexpression PC9/ER cells treated with osimertinib (1 μmol) for 48 hours (D). The histogram shows the different apoptotic rates (E). F, G. Transwell assays show the decreased migration and invasion capacity in miR-764 overexpression PC9/ER cells (F). Scare bar, 200 μm. The histogram shows the numbers of migration and invasion cells (G).

To identify the role of miR-764 in osimertinib resistance, we transfected mimic-miR-764 in PC9/ER cells and exposed cells with osimertinib; we found that the IC50 values decreased in mimic-miR-764 cells (Figure 5A). Subsequently, we observed characteristic bubbles in miR-764 overexpression PC9/ER cells treated with osimertinib (Figure 5B). GSDME, caspase-3, and cleaved caspase-3 were upregulated in miR-764 overexpression PC9/ER cells treated with osimertinib (Figure 5C). Additionally, there was a higher apoptotic rate in miR-764 overexpression PC9/ER cells treated with osimertinib than in those transfected with mimic-control (Figure 5D, E). We also found that miR-764 overexpression can decrease the migration and invasion ability, which indicates that miR-764 can inhibit tumor metastasis (Figure 5F, G). These results revealed that miR-764 promoted pyroptosis and apoptosis, and decreased osimertinib resistance and metastasis.

### MAPK1 is targeted by the circ7312/miR-764 axis

**Figure 6:**
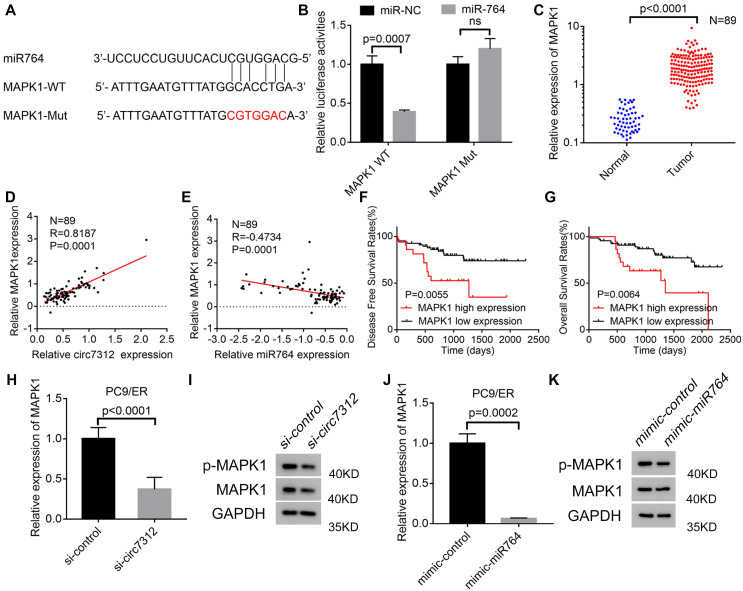
MAPK1 targeted by the circ7312/miR-764 axis. A. The sequence of the miR-764 and MAPK1 fragment including the potential binding site is predicted and synthesized. B. Luciferase assays clarify the correlation between miR-764 and MAPK1. C. The expression of MAPK1 is tested using qualitative real-time polymerase chain reaction (qRT-PCR) in 89 lung adenocarcinoma tissues and paired normal tissues. D, E. Spearman correlation analysis shows the positive correlation between circ7312 and MAPK1 (D) and the negative correlation between miR-764 and MAPK1 (E). F, G. Kaplan-Meier survival analysis clarifies the disease-free survival (F) and overall survival (G) between the high MAPK1 expression and low MAPK1 expression groups. H, I. The relative expression of MAPK1 is tested in PC9/ER cells with circ7312 knockdown for 48 hours by qRT-PCR (H) and Western blotting (I). J-K. The relative expression of MAPK1 is tested in PC9/ER cells with miR-764 overexpression for 48 hours by qRT-PCR (J) and Western blotting (K).

We determined that miR-764 can interact with MAPK1 using a bioinformatics tool. To identify the interaction between miR-764 and MAPK1, we predicted the potential binding site between miR-764 and MAPK1 (Figure 6A). MAPK1-WT increased luciferase activity, and this effect was inhibited by MAPK1-MUT (Figure 6B). To clarify the potential role of MAPK1 in LUAD, we detected the relative expression of MAPK1in 89 LUAD tumor tissues and paired normal tissues using qRT-PCR, indicating that MAPK1 was highly expressed in tumor tissues (Figure 6C). We also confirmed a positive correlation between circ7312 and MAPK1 (Figure 6D) and a negative correlation between miR-764 and MAPK1 (Figure 6E). Kaplan-Meier analysis and log-rank tests showed that high MAPK1 expression significantly decreased DFS and OS (Figure 6F, G). Furthermore, we detected MAPK1 in circ7312 knockdown cells and miR-764 overexpressed cells (Figure 6H-K). These results suggest that circ7312 can sponge miR-764 to regulate MAPK1 expression.

### Circ7312/miR-764/MAPK1 axis promotes osimertinib resistance and reduces pyroptosis and apoptosis

**Figure 7:**
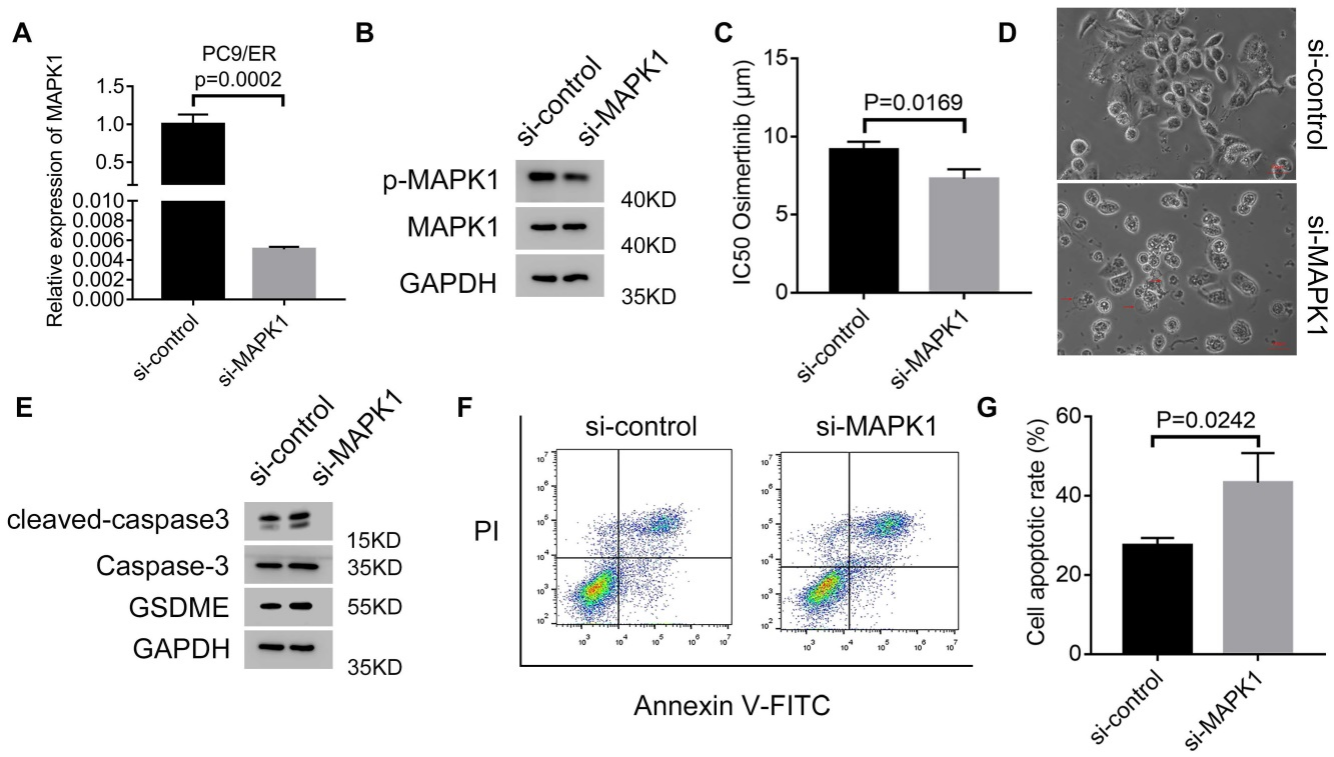
The Circ7312/miR-764/MAPK1 axis promotes osimertinib resistance and reduces pyroptosis and apoptosis. A, B. The transfection efficiency of small interfering RNA targeting MAPK1(si-MAPK1) is tested after 48 hours by qualitative real-time polymerase chain reaction (A) and Western blotting (B). C. Half-maximal inhibitory concentration (IC50) values of osimertinib in MAPK1 knockdown PC9/ER cells treated with osimertinib for 48 hours are identified by Cell Counting Kit-8 assays. D. Representative images of dying MAPK1 knockdown PC9/ER cells treated with osimertinib (1 μmol) for 12 hours. The red arrows indicate cells swelling with large bubbles. Scare bar, 100 μm. E. Western blotting analysis of GSDME, caspase-3, and cleaved caspase-3 protein expressions in MAPK1 knockdown PC9/ER cells treated with osimertinib (1 μmol) for 48 hours. F, G. The apoptotic rate is measured using Annexin V-FITC/PI double staining in MAPK1 knockdown PC9/ER cells treated with osimertinib (1 μmol) for 48 hours (F). The histogram shows the different apoptotic rates (G).

To clarify whether MAPK1 plays a vital role in osimertinib resistance, we transfected si-MAPK1 in PC9/ER cells, and transfection efficiency was assessed by qRT-PCR and Western blotting (Figure 7A, B). The IC50 values of osimertinib were inhibited by MAPK1 knockdown in PC9/ER cells treated with osimertinib (Figure 7C). Additionally, we observed characteristic bubbles in MAPK1 knockdown PC9/ER cells treated with osimertinib (Figure 7D), and Western blotting results indicated that the expressions of GSDME, caspase-3, and cleaved caspase-3 increased in MAPK1 knockdown PC9/ER cells treated with osimertinib (Figure 7E). Furthermore, apoptotic assays showed a higher apoptotic rate in MAPK1 knockdown PC9/ER cells treated with osimertinib (Figure 7F, G). All these data illustrated that the circ7312/miR-764/MAPK1 axis is involved in osimertinib resistance by regulating pyroptosis and apoptosis.

### Targeting circ7312 inhibits tumor growth and osimertinib resistance *in vivo*

**Figure 8:**
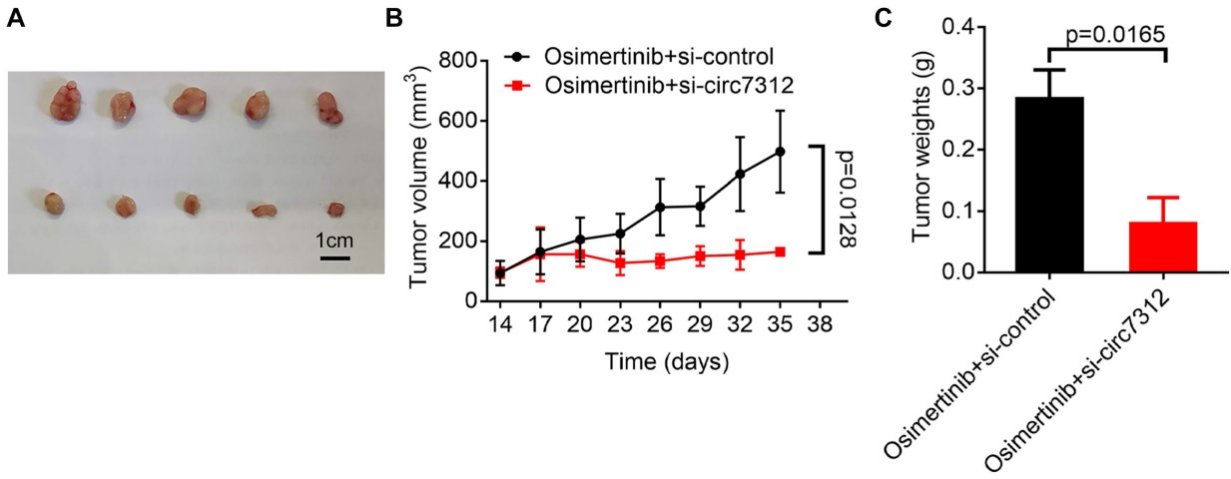
Targeting circ7312 inhibits tumor growth and osimertinib resistance *in vivo*. A. Representative xenograft images of the small interfering RNA negative control (si-control) treatment and small interfering RNA targeting circ-0007312 (si-circ7312) treatment groups. Scare bar, 1 cm. B. Growth curve of the si-control treatment and si-circ7312 treatment groups. C. Histogram of tumor weights of the si-control treatment and si-circ7312 treatment groups.

To further clarify the role of circ7312 *in vivo*, we subcutaneously injected PC9/ER cells into the back of female BALB/C nude mice and divided them into two groups when the tumor grew to approximately 100 mm^3^. We found that the tumor volume was significantly reduced in the group with si-circ7312 treatment compared to the control group (Figure 8A). As shown in Figure 8B, C, targeting circ7312 inhibited tumor growth and osimertinib resistance.

## Discussion

Although osimertinib improved first-generation EGFR-TKI resistance, osimertinib resistance is still inevitable 23. In the current study, we found that circ7312 participates in osimertinib resistance by the miR-764/MAPK1 axis, which provides new insight into osimertinib resistance in LUAD. We also demonstrated that pyroptosis and apoptosis are involved in osimertinib resistance; therefore, we further explored the role of the circ7312/miR-764/MAPK1 axis in pyroptosis and apoptosis, which are involved in osimertinib resistance and thus confirms our hypothesis that pyroptosis is involved in osimertinib resistance.

Recently, pyroptosis was identified as a new non-inflammatory PCD that participates in progression of various tumors, including digestive, breast, and lung cancers. Some dying cells displayed typical morphology of pyroptosis, such as cell swelling, bubble formation, and osmotic lysis 24. We observed large bubbles on the membrane in PC9 cells treated with osimertinib rather than PC9/ER cells. Therefore, we speculated that pyroptosis was involved in osimertinib resistance. Chemotherapy drug-induced pyroptosis can be triggered by cleaved GSDMD, which is activated by the classic caspase-1 pathway or non-classic caspase-4/5/11 pathway. Besides, pyroptosis can also be triggered by cleaved GSDME, which was activated by caspase-3 13. In addition, caspase-1-associated circRNA was reported to act as an miR-214-3p sponge to activate the caspase-1 dependent pathway to trigger pyroptosis in diabetic cardiomyopathy, and previous studies clarified that circRNAs play a vital role in pyroptosis 25. Nevertheless, the underlying mechanisms of pyroptosis induced by EGFR-TKIs is unclear, so we explored the underlying mechanisms and found that circ7312 knockdown, miR-764 overexpression, and MAPK1 knockdown can increase the GSDME protein expression. We also observed large bubbles on the membrane in treated PC9/ER cells, indicating that circ7312 knockdown, miR-764 overexpression, and MAPK1 knockdown can decrease osimertinib resistance by promoting pyroptosis.

Apoptosis is the most studied PCD form that can maintain the survival/death balance in cells, and the dysregulation of apoptotic signals is involved in tumorigenesis and tumor progression 26, 27. Many anti-cancer drugs trigger cancer cell death by activating apoptotic signaling pathways, but the dysregulation of apoptotic pathways can result in drug resistance 28. Therefore, exploring the underlying mechanisms of apoptosis in drug resistance is urgently needed. Many studies have demonstrated that apoptosis is involved in EGFR-TKI resistance; for example, FGL1 can mediate gefitinib resistance by reducing apoptosis in non-small cell lung cancer, and Yiqi Chutan Tang can target apoptosis and autophagy to relieve gefitinib resistance 29, 30. In our study, we found that circ7312 knockdown, miR-764 overexpression, and MAPK1 knockdown increased the apoptotic rate in treated PC9/ER cells, clarifying that circ7312 knockdown, miR-764 overexpression, and MAPK1 knockdown decrease osimertinib resistance by promoting apoptosis.

CircRNAs are characterized by a covalently closed structure with neither 5'caps nor 3'trials. Increasing evidence has demonstrated that circRNAs participate in tumorigenesis and progression in various tumors, such as lung, gastric, and cervical cancers 31-33. Additionally, circRNAs are involved in drug resistance. For instance, circ-CPA4 was identified to regulate drug resistance via the let-7 miRNA/PD-L1 axis, and circ_PIP5K1A was considered to regulate cisplatin sensitivity through the miR‑101/ABCC1 axis 17, 34. However, the role of circRNAs in osimertinib resistance remains elusive. We found that circ7312 knockdown decreased osimertinib resistance and increased pyroptosis and apoptosis, indicating that circ7312 may promote osimertinib resistance by pyroptosis and apoptosis. Furthermore, we also found that circ7312 was highly expressed in tumor tissue and was associated with a poor prognosis. Xenograft experiments revealed that targeting circ7312 can decrease osimertinib resistance and inhibit tumor growth.

Emerging studies have demonstrated that miRNAs regulate oncogenic or tumor suppressor signaling pathways to exert influence in the development of cancer, and miRNAs are also implicated in drug resistance by regulating physiological processes, such as apoptosis, cell cycle control, and DNA damage repair 35. Zhu et al. reported that miR-764 sponged by long non-coding RNA Mirt2 reduced apoptosis in acute myocardial infarction 36. Ding et al. clarified that miR-764 regulates hydrogen peroxide-induced neuronal cell death 37. Chen et al. reported that miR-764 expression in plasma is relevant to the prognosis of hepatocellular carcinoma, indicating the potential of miR-764 as a biomarker for hepatocellular carcinoma 38. In this study, we found that miR-764 can be negatively regulated by circ7312. Luciferase assays indicated that circ7312 directly binds to miR-764, and circ7312 knockdown can also increase the expression of miR-764. Spearman correlation analysis confirmed a negative correlation between circ7312 and miR-764. These studies showed that miR746 is involved in cell death, suggesting that miR-764 may promote osimertinib resistance.

MAPK1 is a key protein that regulates the Ras-Raf-MEK-ERK signaling pathway, which can mediate various physiological and pathological processes, including cell apoptosis, proliferation, differentiation, and immune response^39^. The ERK/MAPK signaling pathway is also related to tumorigenesis and metastasis; increased ERK expression was found in various tumors, e.g., lung, breast, and colon cancers, and the MAPK signaling pathway modulates drug resistance 40, 41. In our study, we found that MAPK1 knockdown can decrease osimertinib resistance and enhance pyroptosis and apoptosis, and MAPK1 was highly expressed in tumor tissues and related to prognosis in LUAD. Moreover, we found that circ7312 knockdown and miR-764 overexpression inhibited the expression of MAPK1, and Spearman correlation analysis indicated a positive correlation between circ7312 and MAPK1 and a negative correlation between miR-764 and MAPK1. Therefore, we demonstrated that circ7312 can promote osimertinib through the miR-764/MAPK1 axis.

In conclusion, our study identified that the circ7312/miR-764/MAPK1 axis can promote osimertinib resistance by regulating pyroptosis and apoptosis. Moreover, circ7312 and MAPK1 were highly expressed in tumor tissues and associated with poor prognosis. We also found that circ7312 knockdown decreased osimertinib resistance and tumor growth *in vivo*. Our study provides a novel therapeutic target for treating advanced LUAD with osimertinib resistance.

## Supplementary Material

Supplementary table.

## Figures and Tables

**Table 1:** The correlation between circ7312 expression and clinical pathological feature (n=89)

Clinical feature	Number	circ7312 expression		*P* value
		High expression	Low expression	
Total number	89	44	45	
Age(years)				
≤60	56	32	24	0.0582
>60	33	12	21	
Gender				
Male	50	27	23	0.3297
Female	39	17	22	
Smoking history				
No	45	19	26	
Yes	44	25	19	
TNM stage				
Ⅰ/Ⅱ	62	17	45	<0.0001
Ⅲ	27	27	0	
5-years survival				
NO	80	35	45	0.0044
YES	9	9	0	

Bold font *P<0.05*
